# Supplementary material for: Molecular basis of egg coat cross-linking sheds light on ZP1-associated female infertility
Source: Nat Commun. 2019 Jul 12;10:3086. doi: 10.1038/s41467-019-10931-5 (PMC6626044; doi:10.1038/s41467-019-10931-5)
Supplement: Supplementary file 1 — Supplementary Information [file 41467_2019_10931_MOESM1_ESM.pdf]

Supplementary Information

Molecular basis of egg coat cross-linking sheds light on ZP1-associated female infertility

Kaoru Nishimura[1], Elisa Dioguardi[1], Shunsuke Nishio[2,3], Alessandra Villa[1], Ling Han[1], Tsukasa Matsuda[2] & Luca Jovine[1]

[1] Department of Biosciences and Nutrition and Center for Innovative Medicine, Karolinska Institutet, Huddinge, SE-141 83, Sweden

[2] Department of Applied Molecular Biosciences, Graduate School of Bioagricultural Sciences, Nagoya University, Chikusa, Nagoya, Japan

[3] Present address: Department of Biosciences and Nutrition and Center for Innovative Medicine, Karolinska Institutet, Huddinge, SE-141 83, Sweden

These authors contributed equally: Kaoru Nishimura, Elisa Dioguardi

Correspondence and requests for materials should be addressed to Luca Jovine (luca.jovine@ki.se)

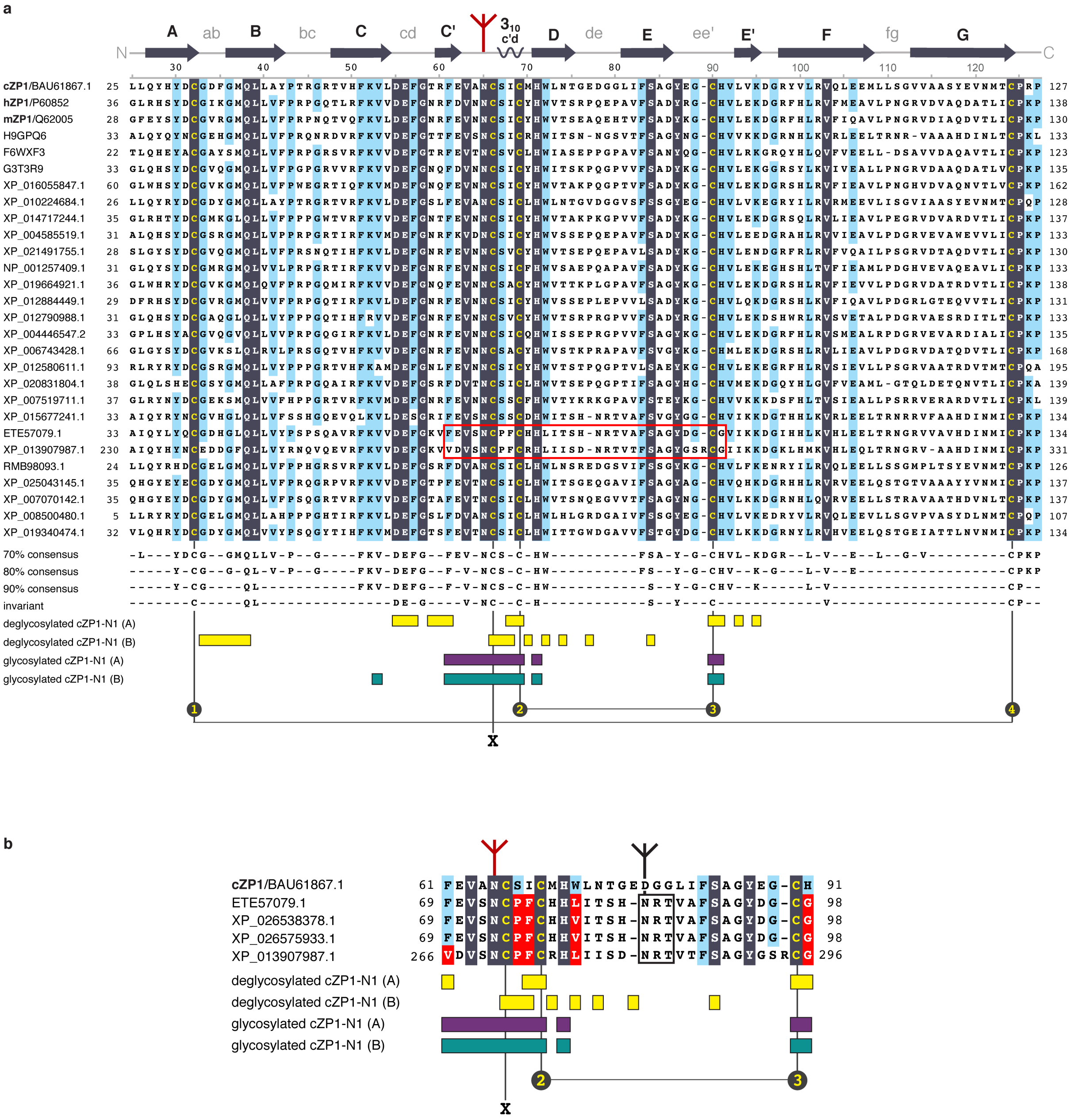
a
N
A
ab
B
bc
C
cd
C'
3₁₀
c'd
D
de
E
ee'
E'
F
fg
G
C
cZP1/BAU61867.1
hZP1/P60852
mZP1/Q62005
H9GPQ6
F6WXF3
G3T3R9
XP_016055847.1
XP_010224684.1
XP_014717244.1
XP_004585519.1
XP_021491755.1
NP_001257409.1
XP_019664921.1
XP_012884449.1
XP_012790988.1
XP_004446547.2
XP_006743428.1
XP_012580611.1
XP_020831804.1
XP_007519711.1
XP_015677241.1
ETE57079.1
XP_013907987.1
RMB98093.1
XP_025043145.1
XP_007070142.1
XP_008500480.1
XP_019340474.1
70% consensus
80% consensus
90% consensus
invariant
deglycosylated cZP1-N1 (A)
deglycosylated cZP1-N1 (B)
glycosylated cZP1-N1 (A)
glycosylated cZP1-N1 (B)
X
b
cZP1/BAU61867.1
ETE57079.1
XP_026538378.1
XP_026575933.1
XP_013907987.1
deglycosylated cZP1-N1 (A)
deglycosylated cZP1-N1 (B)
glycosylated cZP1-N1 (A)
glycosylated cZP1-N1 (B)
X

Supplementary Figure 1. Sequence alignment of representative ZP1-N1 sequences.

a, Alignment of a 80% identity non-redundant ZP1-N1 sequence database, with invariant residues shaded in dark blue and amino acids above the 80% identity cut-off shaded in cyan. Cys residues are highlighted in yellow. Sequences are indicated by accession numbers and associated amino acid boundaries. Above the alignment, the residue numbering and secondary structure diagram of cZP1 are reported. β-strands are indicated by arrows, whereas the 3_{10} helix in the c'd loop (only found in chain A of deglycosylated cZP1-N1 as well as chain B of the glycosylated protein) is depicted as a squiggle. An inverted dark red tripod symbolizes the glycan attached to the highly conserved N-glycosylation site of ZP1-N1. Below the alignment, residues matching different identity thresholds within the 100%-70% range are shown, and positions corresponding to amino acids involved in the different cZP1-N1 homodimer interfaces are indicated by bars, coloured according to Fig. 5. The canonical ZP-N intramolecular disulphide pattern is also shown, and the ZP1-specific invariant Cys forming the intermolecular cross-link is indicated by the X symbol.

b, Expansion of the alignment region contained within the red rectangle of panel a to include all members from two clusters consisting of sequences from the suborder Serpentes. The corresponding region of the cZP1 sequence is also reported for comparison, and elements are represented as in panel a. Notable variations in homodimer interface residues are highlighted using red shading. A black rectangle marks the sequon that replaces the N-glycosylation site corresponding to cZP1 N65 (inverted dark red tripod) with one located in the de loop (inverted black tripod).

a

b

c

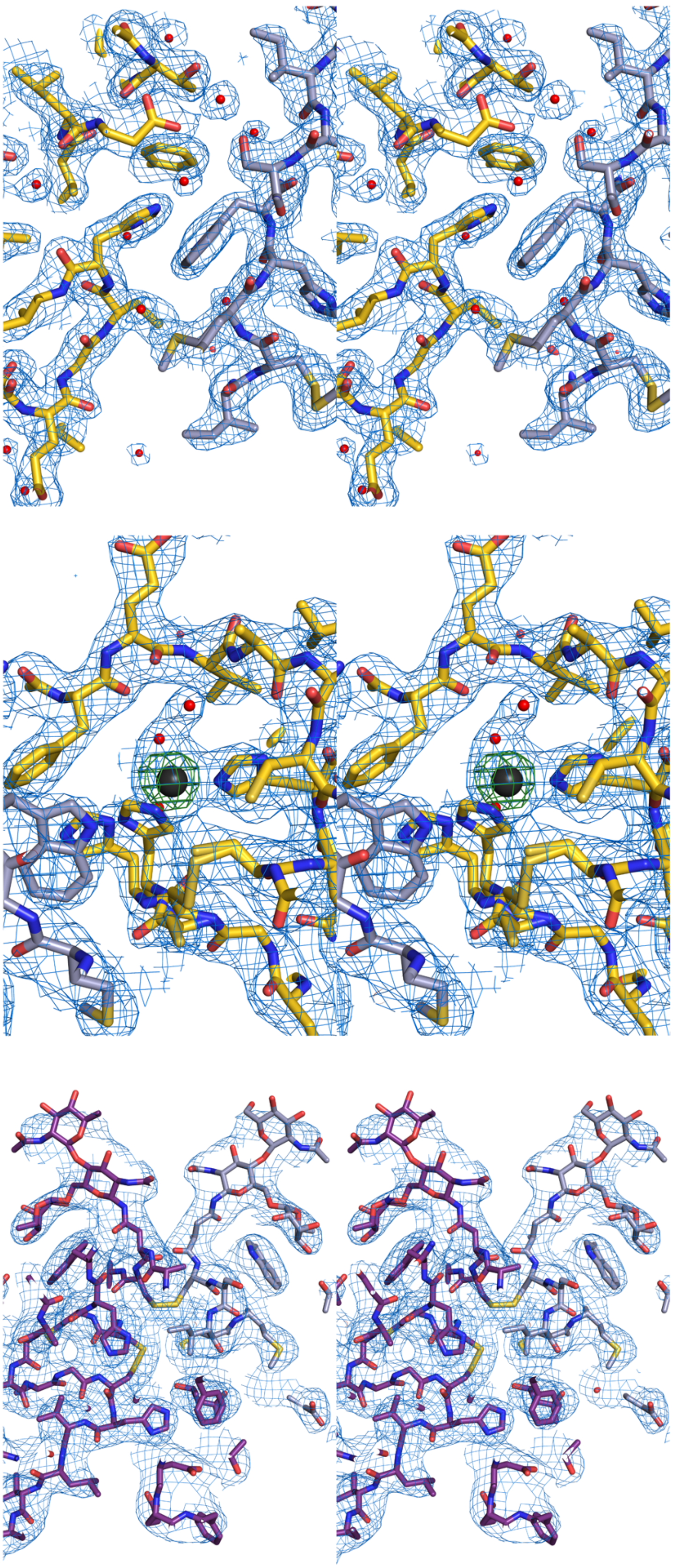

Supplementary Figure 2. Representative sections of the electron density maps of cZP1-N1.

Walleye stereo maps are superimposed onto the corresponding atomic models, shown in stick representation and coloured as in Fig. 8.

a, *2mFo-DFc* electron density maps of Endo H-treated cZP1-N1 (PDB ID 6GF6), contoured at 1 σ and centred around H91(A) and W72(B) at the homodimer interface.

b, *2mFo-DFc* (blue; contoured at 1 σ) and anomalous difference map at λ=1.2825 Å (green; calculated and contoured at 6 σ) of the Endo-H treated cZP1-N1 crystal soaked with $Zn(OAc)_2$ (PDB ID 6GF7), centred around the Zn^{2+}-binding site.

c, *2mFo-DFc* electron density maps of glycosylated cZP1-N1 (PDB ID 6GF8), contoured at 1 σ and centred around the C66-C66 intermolecular disulphide.

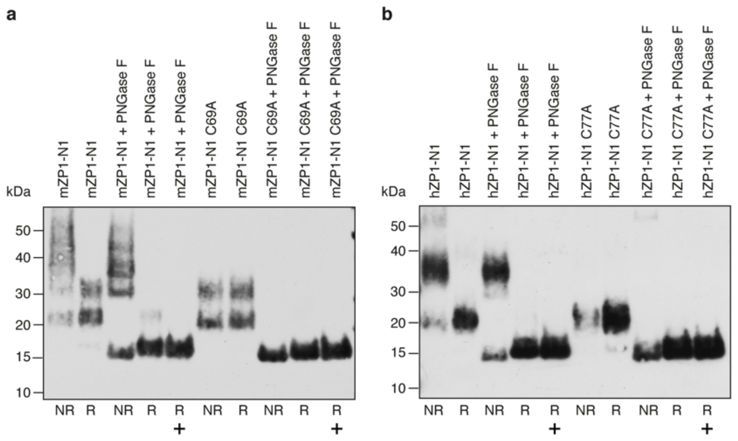

Supplementary Figure 3. Deglycosylation of mouse and human ZP1-N1 domains.

a-b, Anti-5His immunoblot analysis of purified mZP1-N1 (panel a) and hZP1-N1 (panel b) proteins before and after deglycosylation with PNGase F.

The experiment was performed using the material shown in Fig. 6e, g, and samples digested using the manufacturer's Glycoprotein Denaturing Buffer, which contains DTT, are indicated by a plus sign. Note that PNGase F is unable to efficiently deglycosylate the homodimeric form of the mammalian proteins in non-reducing conditions; this suggests that, like cZP1 N65, N76 of hZP1 and N68 of mZP1 also lie in close proximity to the cross-link interface.

a

MGRSRSLLLP LLLLLPAGLP SGLALLQYHY DCGDFGMQLL AYPTRGRTVH FKVLDEFGTR FEVANCSICM
HWLNTGEDGG LIFSAGYEGC HVLVKDGRYV LRVQLEEMLL SGVVAASYEV NMTCPRPAGY EILRDEKVHG
HQRPDRGNGA LSSHGVNVLI PRPRPGLLQH TAHSALAIPR PQLPPGAPVE QSHSMAHTQS

Start	–	End	Observed	Mr (expt)	Mr (calc)	Delta	M	Score	Peptide
29	–	35	913.2820	912.2748	912.3072	-0.0325	1	43	Y.HYDCGDF.G
29	–	40	1471.5972	1470.5899	1470.5908	-0.0009	3	65	Y.HYDCGDFGMQLL.A + Oxidation (M)
41	–	51	1304.6818	1303.6745	1303.6786	-0.0040	2	19	L.AYPTRGRTVHF.K
52	–	61	1211.6322	1210.6249	1210.6346	-0.0097	4	59	F.KVLDEFGTRF.E
84	–	93	1092.4794	1091.4721	1091.4706	0.0015	1	52	F.SAGYEGCHVL.V
84	–	99	1810.8734	1809.8661	1809.8468	0.0193	4	74	F.SAGYEGCHVLVKDGRY.V
100	–	110	1342.7635	1341.7562	1341.7690	-0.0128	4	61	Y.VLRVQLEEMLL.S
100	–	110	1358.7531	1357.7458	1357.7639	-0.0181	4	13	Y.VLRVQLEEMLL.S + Oxidation (M)
131	–	146	1985.0438	1984.0365	1984.0351	0.0014	3	46	Y.EILRDEKVHGHQRPDR.G
135	–	151	1886.9166	1885.9093	1885.9143	-0.0050	2	63	R.DEKVHGHQRPDRGNGAL.S + Deamidated (NQ)
138	–	151	1514.7451	1513.7378	1513.7498	-0.0120	1	58	K.VHGHQRPDRGNGAL.S + Deamidated (NQ)
152	–	168	1813.0298	1812.0225	1812.0370	-0.0145	2	81	L.SSHGVNVLIPRPRPGLL.Q + Deamidated (NQ)
177	–	195	2012.0358	2011.0285	2011.0309	-0.0024	0	98	L.AIPRPQLPPGAPVEQSHSM.A
177	–	195	2028.0425	2027.0352	2027.0258	0.0094	0	78	L.AIPRPQLPPGAPVEQSHSM.A + Oxidation (M)

b

MGRSRSLLLP LLLLLPAGLP SGLALLQYHY DCGDFGMQLL AYPTRGRTVH FKVLDEFGTR FEVANCSICM
HWLNTGEDGG LIFSAGYEGC HVLVKDGRYV LRVQLEEMLL SGVVAASYEV NMTCPRPAGY EILRDEKVHG
HQRPDRGNGA LSSHGVNVLI PRPRPGLLQH TAHSALAIPR PQLPPGAPVE QSHSMAHTQS

Start	–	End	Observed	Mr (expt)	Mr (calc)	Delta	M	Score	Peptide
29	–	35	913.2836	912.2764	912.3072	-0.0309	2	40	Y.HYDCGDF.G
29	–	39	1342.5234	1341.5161	1341.5118	0.0043	4	78	Y.HYDCGDFGMQL.L
29	–	40	1455.5782	1454.5709	1454.5959	-0.0250	5	65	Y.HYDCGDFGMQLL.A
29	–	40	1455.6118	1454.6045	1454.5959	0.0086	5	57	Y.HYDCGDFGMQLL.A
41	–	50	1157.5958	1156.5885	1156.6101	-0.0216	1	28	L.AYPTRGRTVH.F
41	–	51	1304.6456	1303.6383	1303.6786	-0.0402	2	30	L.AYPTRGRTVHF.K
52	–	61	1211.6364	1210.6291	1210.6346	-0.0055	2	69	F.KVLDEFGTRF.E
52	–	70	2277.0176	2276.0103	2276.0276	-0.0172	3	31	F.KVLDEFGTRFEVANCSICM.H + Deamidated (NQ)
71	–	83	1458.6865	1457.6792	1457.6939	-0.0147	4	90	M.HWLNTGEDGGLIF.S
100	–	110	1342.7843	1341.7770	1341.7690	0.0080	4	70	Y.VLRVQLEEMLL.S
100	–	110	1358.7571	1357.7498	1357.7639	-0.0141	4	24	Y.VLRVQLEEMLL.S + Oxidation (M)
119	–	130	1395.5591	1394.5518	1394.5959	-0.0440	1	57	Y.EVNMTCPRPAGY.E + Deamidated (NQ)
119	–	130	1395.6145	1394.6072	1394.5959	0.0114	1	35	Y.EVNMTCPRPAGY.E + Deamidated (NQ)
119	–	130	1411.5787	1410.5714	1410.5908	-0.0194	1	21	Y.EVNMTCPRPAGY.E + Oxidation (M); Deamidated (NQ)
123	–	130	921.4077	920.4004	920.4174	-0.0170	0	32	M.TCPRPAGY.E
131	–	146	1985.0028	1983.9955	1984.0351	-0.0396	3	66	Y.EILRDEKVHGHQRPDR.G
135	–	151	1886.8840	1885.8767	1885.9143	-0.0376	2	60	R.DEKVHGHQRPDRGNGAL.S + Deamidated (NQ)
140	–	151	1278.6187	1277.6114	1277.6225	-0.0111	1	42	H.GHQRPDRGNGAL.S + Deamidated (NQ)
152	–	168	1813.0149	1812.0076	1812.0370	-0.0294	3	79	L.SSHGVNVLIPRPRPGLL.Q + Deamidated (NQ)
152	–	168	1813.0236	1812.0163	1812.0370	-0.0207	3	88	L.SSHGVNVLIPRPRPGLL.Q + Deamidated (NQ)
169	–	176	864.4077	863.4004	863.4250	-0.0245	2	41	L.QHTAHSAL.A
177	–	193	1793.9420	1792.9347	1792.9584	-0.0237	1	44	L.AIPRPQLPPGAPVEQSH.S
177	–	195	2012.0015	2010.9942	2011.0309	-0.0367	2	108	L.AIPRPQLPPGAPVEQSHSM.A
177	–	195	2028.0200	2027.0127	2027.0258	-0.0131	2	11	L.AIPRPQLPPGAPVEQSHSM.A + Oxidation (M)
177	–	195	2028.0365	2027.0292	2027.0622	-0.0330	2	68	L.AIPRPQLPPGAPVEQSHSM.A + Oxidation (M); Deamidated (NQ)

c

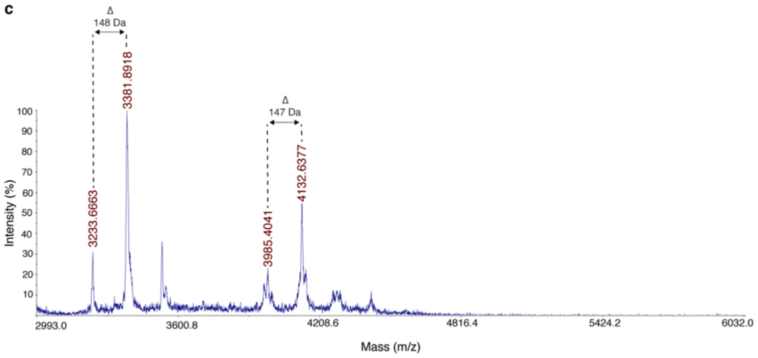

Supplementary Figure 4. Mass spectrometry analysis of native cZP1 N-terminal fragment.

a-b, Soluble material released by sperm protease treatment of native chicken egg coats was reduced, digested with chymotrypsin and subjected to LC-MALDI-TOF MS/MS analysis. Sequence coverage by MS analysis is indicated in red and N-glycosylation sites are underlined. Whereas cZP1 N-terminal fragment peptides including N65 or N121 are missing in the fully glycosylated sample (panel a), the material treated with PNGase F shows peptides containing N65 or N121 (panel b, grey highlight).

c, Chymotryptic glycopeptides were enriched using RCA-1 lectin and analysed by MALDI-TOF MS analysis. Four major peaks above 3000 m/z were detected: the first two (3233.6663 and 3381.3193 m/z) are consistent with a glycopeptide containing the N-glycan linked to N121 of the peptide E119-Y130 (Mr, 1396), whereas the 3985.4041 and 4132.6377 m/z peaks are consistent with a peptide carrying a carbohydrate linked to N65 of the peptide K52-M70 (Mr, 2277). Comparison of these pairs of peaks suggests that they differ by a single fucose residue (mass 146 Da).

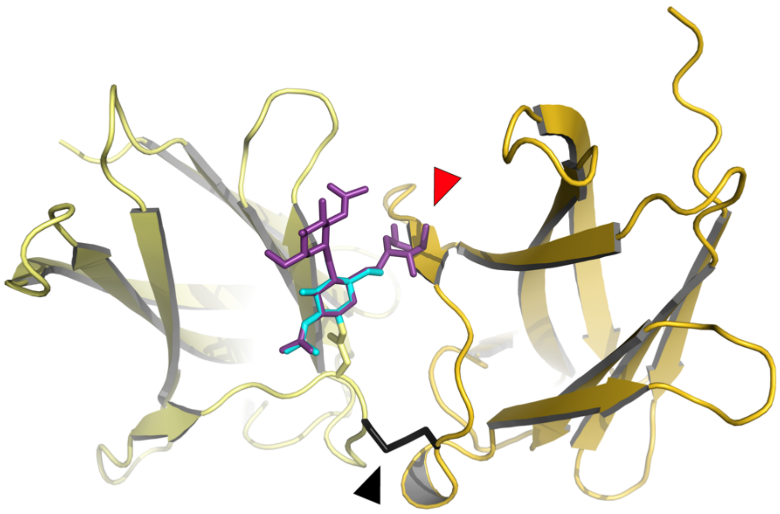

Supplementary Figure 5. Superimposition of the N65-linked glycans of glycosylated and Endo H-treated cZP1-N1.

Chains A and B of Endo H-treated cZP1-N1 are depicted in cartoon representation and coloured gold and yellow, respectively; the C66 cross-link (black arrow), as well as N65(B) and the trimmed carbohydrate attached to it, are shown as sticks. The core GlcNac residue of the N65-linked carbohydrate of glycosylated cZP1-N1 (violet purple) is superimposed onto the corresponding GlcNac of the Endo H-treated protein (cyan). As indicated by a red arrow, presence of an α1-6-linked fucose in the glycan attached to N65(B) would not be compatible with the asymmetric conformation of the cZP1-N1 homodimer, because it would clash with β-strand C' of chain A of the latter.

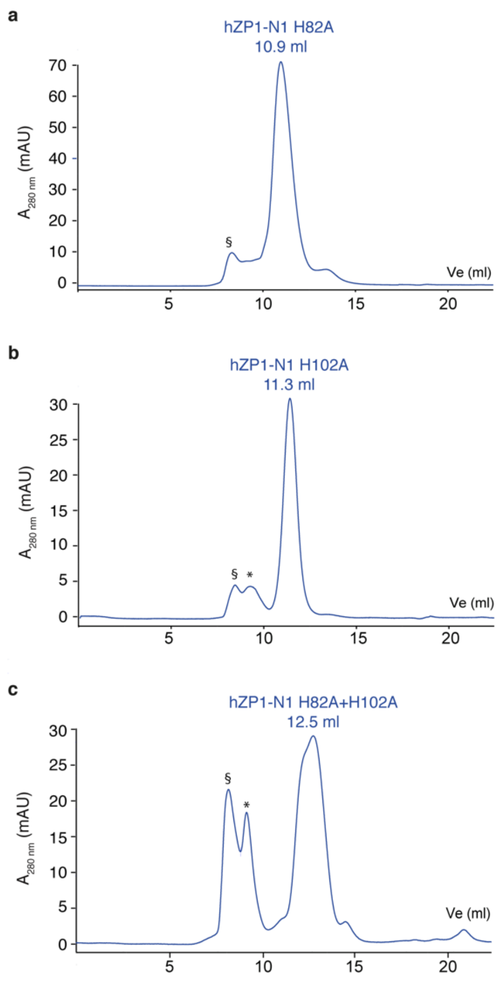

Supplementary Figure 6. SEC Analysis of hZP1-N1 mutants.

a-c, SEC profiles of hZP1-N1 mutants H82A (panel a), H102A (panel b) and hZP1-N1 H82A+H102A (panel c) using a Superdex 75 Increase 10/300 GL column. A Coomassie-stained SDS-PAGE analysis of the corresponding peaks fractions is shown in Fig. 8h. Elution volumes are reported; void volume and contaminant peaks are indicated by § and *, respectively.

Supplementary Table 1 Marsupial and eutherian ZP properties

	ZP composition				ZP thickness
	ZP1	ZP2	ZP3	ZP4	
Gray short tail opussum[87] *(Monodelphis domestica)*	+	+	pseudogene ZP3a (ZP3b, c)	pseudogene	1.6-2.0[88] µm 0.5 µm -1 µm[89] (no more than 4 µm[90–92])
Brushtail possum *(Trichosurus vulpecula)*	+	+	+	+	8 µm[93]
Tammar Wallaby[87] *(Macropus eugenii)*	+	+	(ZP3a, b, c)	+	4-8.5 µm[93]
Mouse (*Mus musculus*)	+	+	+	*pseudogene*	5-7 µm[94]
Hamster *(Mesocricetus auratus)*	+	+	+	+	10-16 µm[95]
Human *(Homo sapiens)*	+	+	+	+	15-20 µm[21]
Rabbit *(Oryctolagus cuniculus)*	+	+	+	+	16 µm[96]
Cat *(Felis catus)*	+	+	+	+	15 µm[97]
Dog *(Canis lupus)*	-	+	+	+	10-15 µm[98]
Pig *(Sus scrofa)*	-	+	+	+	16 µm[99]
Cow *(Bos taurus)*	-	+	+	+	27 µm[99]
Horse *(Equus caballus)*	+	+	+	+	8 µm[97]
