# Supplementary material for: Molecular basis of egg coat cross-linking sheds light on ZP1-associated female infertility
Source: Nat Commun. 2019 Jul 12;10:3086. doi: 10.1038/s41467-019-10931-5 (PMC6626044; doi:10.1038/s41467-019-10931-5)
Supplement: Supplementary file 2 — Reporting Summary [file 41467_2019_10931_MOESM2_ESM.pdf]

nature research

Corresponding author(s): Luca Jovine

Reporting Summary

Nature Research wishes to improve the reproducibility of the work that we publish. This form provides structure for consistency and transparency in reporting. For further information on Nature Research policies, see Authors & Referees and the Editorial Policy Checklist.

Statistical parameters

When statistical analyses are reported, confirm that the following items are present in the relevant location (e.g. figure legend, table legend, main text, or Methods section).

n/a	Confirmed	
☐	☒	The exact sample size (*n*) for each experimental group/condition, given as a discrete number and unit of measurement
☐	☒	An indication of whether measurements were taken from distinct samples or whether the same sample was measured repeatedly
☒	☐	The statistical test(s) used AND whether they are one- or two-sided<br>*Only common tests should be described solely by name; describe more complex techniques in the Methods section.*
☒	☐	A description of all covariates tested
☒	☐	A description of any assumptions or corrections, such as tests of normality and adjustment for multiple comparisons
☒	☐	A full description of the statistics including central tendency (e.g. means) or other basic estimates (e.g. regression coefficient) AND variation (e.g. standard deviation) or associated estimates of uncertainty (e.g. confidence intervals)
☒	☐	For null hypothesis testing, the test statistic (e.g. *F*, *t*, *r*) with confidence intervals, effect sizes, degrees of freedom and *P* value noted<br>*Give P values as exact values whenever suitable.*
☒	☐	For Bayesian analysis, information on the choice of priors and Markov chain Monte Carlo settings
☒	☐	For hierarchical and complex designs, identification of the appropriate level for tests and full reporting of outcomes
☒	☐	Estimates of effect sizes (e.g. Cohen's *d*, Pearson's *r*), indicating how they were calculated
☒	☐	Clearly defined error bars<br>*State explicitly what error bars represent (e.g. SD, SE, CI)*

Our web collection on statistics for biologists may be useful.

Software and code

Policy information about availability of computer code

Data collection	All X-ray diffraction datasets were collected from single crystals at 100 K, using either a MarMosaic 225 CCD (Mar) (Endo H-treated cZP1-N1 native data) or PILATUS 200K or 6M/6M-F detectors (DECTRIS) (all other datasets). Glycosylated cZP1-N1 datasets were collected at European Synchrotron Radiation Facility (ESRF, Grenoble) beamlines ID2935 (λ=0.9763 Å) and ID23-136 (λ=0.9791 Å), respectively. Endo H-treated cZP1-N1 datasets were collected at beamline BL14.1 of BESSY II (Helmholtz-Zentrum, Berlin) (native data, λ=1.7710 Å) and beamline I03 of Diamond Light Source (DLS, England) (gold derivative data, λ=1.03970 Å; zinc derivative data; λ=1.2825 Å).<br>For nano-LC/MS analysis, we used a DiNa Nano LC system equipped with a DiNa MaP autospotter (KYA technologies); MS spectra were acquired using a MALDI-TOF/TOF 5800 Proteomic Analyzer mass spectrometer (Applied Biosystems).
Data analysis	X-ray structure determination was carried out using XDS (PMID 20124692), Phaser (PMID 19461840), PHENIX AutoSol (PMID 19465773), PHENIX AutoBuild (PMID 18094468), Coot (PMID 20383002) and phenix.refine (PMID 22505256). Structure validation was performed using MolProbity (PMID 20057044) and Privateer (PMID 26581513). Structural alignments were performed using Chimera (PMID 15264254), Coot and PyMOL (Schrödinger, LLC). Protein-protein interfaces and oligomeric states were analyzed using PISA (PMID 17681537), PIC (PMID 17584791), PDBsum (PMID 9433130) and FoldX4 (PMID: 15980494). Structural figures were created with PyMOL. MS data was analyzed with Mascot (PMID 10612281).

For manuscripts utilizing custom algorithms or software that are central to the research but not yet described in published literature, software must be made available to editors/reviewers upon request. We strongly encourage code deposition in a community repository (e.g. GitHub). See the Nature Research guidelines for submitting code & software for further information.

Data

Policy information about availability of data

All manuscripts must include a data availability statement. This statement should provide the following information, where applicable:

- Accession codes, unique identifiers, or web links for publicly available datasets
- A list of figures that have associated raw data
- A description of any restrictions on data availability

Atomic coordinates and structure factors have been deposited with the Protein Data Bank under accession codes 6GF6 (Endo H-treated cZP1-N1 homodimer, high resolution native), 6GF7 (Endo H-treated cZP1-N1 homodimer, Zn2+ derivative) and 6GF8 (glycosylated cZP1-N1 homodimer).

Field-specific reporting

Please select the best fit for your research. If you are not sure, read the appropriate sections before making your selection.

☒ Life sciences ☐ Behavioural & social sciences

For a reference copy of the document with all sections, see nature.com/authors/policies/ReportingSummary-flat.pdf

Life sciences

Study design

All studies must disclose on these points even when the disclosure is negative.

Sample size	*Describe how sample size was determined, detailing any statistical methods used to predetermine sample size OR if no sample-size calculation was performed, describe how sample sizes were chosen and provide a rationale for why these sample sizes are sufficient.*
Data exclusions	*Describe any data exclusions. If no data were excluded from the analyses, state so OR if data were excluded, describe the exclusions and the rationale behind them, indicating whether exclusion criteria were pre-established.*
Replication	All transfection (Fig. 1b-d; Fig. 2g-i; Fig. 3a; Fig. 6b,d,f; Fig. 7b; Fig. 8f; Supplementary Fig. 3a-b) and pull-down experiments (Fig. 8g; Fig. 9b,c) were repeated at least three times.
Randomization	N/A
Blinding	N/A

Materials & experimental systems

Policy information about availability of materials

n/a	Involved in the study
☐	☒ Unique materials
☐	☒ Antibodies
☐	☒ Eukaryotic cell lines
☐	☒ Research animals
☒	☐ Human research participants

Unique materials

Obtaining unique materials	All expression constructs, as well as the anti-cZP1 antibody, are freely available without restrictions for use to investigators.

Antibodies

Antibodies used	Anti-5His Penta-His monoclonal antibody, BSA-free (QIAGEN cat. no. 34660); anti-c-Myc monoclonal antibody (clone 9E10; Sigma-Aldrich at. no. M4439); anti-FLAG monoclonal antibody (clone M2; Sigma-Aldrich cat. no. F1804); anti-HA monoclonal antibody (clone HA-7; Sigma-Aldrich cat. no. H3663); goat anti-mouse IgG Fc secondary antibody, HRP-conjugated (Thermo Fisher Scientific cat. no. A16084); horse-anti mouse IgG secondary antibody, HRP-conjugated (Cell Signaling Technology; 7076); anti-cZP1 N-terminal fragment polyclonal antibody (PMID 26106520).
Validation	Antibodies were validated by the manufactures.

Eukaryotic cell lines

Policy information about cell lines

Cell line source(s)	HEK293T: laboratory of Prof. A. Radu Aricescu (University of Oxford, UK; now at the MRC Laboratory of Molecular Biology, Cambridge, UK).<br>HEK293S GnTi-: ATCC cat. no. CRL-3022.
Authentication	Cell line authentication was performed by the sources described above.
Mycoplasma contamination	Each cell line was tested for mycoplasma contamination by the respective source.<br>We confirmed that HEK293T and HEK293S were mycoplasma-free by using a PCR Mycoplasma Test Kit II (Applichem cat. no. A8994).
Commonly misidentified lines<br>(See ICLAC register)	N/A: cell lines HEK293T (CVCL_0063) and HEK293S (CVCL_A784) are not listed in version 8 of the Database of Cross-Contaminated or Misidentified Cell Lines.

Research animals

Policy information about studies involving animals; ARRIVE guidelines recommended for reporting animal research

Animals/animal-derived materials	Animal experiments for obtaining native chicken egg coat material (Fig. 2 and Supplementary Fig. 3) were performed in accordance with the approval of the Committee for Animal Experiments of the Graduate School of Bioagricultural Sciences, Nagoya University (approved number 2016030218). The board members at the time of these experiments were Eiichi Hondo, Fumihiko Horio, Yoshiharu Shimomura, Yoichi Matsuda, Yasushige Ohmori, Daita Nadano, Hisashii Kajimura, Tomoaki Niimi, Takahiro Yamagata.

Method-specific reporting

n/a	Involved in the study
☒	☐ ChIP-seq
☒	☐ Flow cytometry
☒	☐ Magnetic resonance imaging
